# Green Intrusion Detection Systems: A Comprehensive Review and Directions

**DOI:** 10.3390/s24175516

**Published:** 2024-08-26

**Authors:** Swapnoneel Roy, Sriram Sankaran, Mini Zeng

**Affiliations:** 1 School of Computing, University of North Florida, Jacksonville, FL 32224, USA; 2 Center for Cybersecurity Systems and Networks, Amrita Vishwa Vidyapeetham, Amritapuri 690525, Kerala, India; srirams@am.amrita.edu; 3 Computing Sciences, Jacksonville University, Jacksonville, FL 32211, USA; mzeng@ju.edu

**Keywords:** energy optimization, intrusion detection, cybersecurity, anomaly detection

## Abstract

Intrusion detection systems have proliferated with varying capabilities for data generation and learning towards detecting abnormal behavior. The goal of green intrusion detection systems is to design intrusion detection systems for energy efficiency, taking into account the resource constraints of embedded devices and analyzing energy–performance–security trade-offs. Towards this goal, we provide a comprehensive survey of existing green intrusion detection systems and analyze their effectiveness in terms of performance, overhead, and energy consumption for a wide variety of low-power embedded systems such as the Internet of Things (IoT) and cyber physical systems. Finally, we provide future directions that can be leveraged by existing systems towards building a secure and greener environment.

## 1. Introduction

In the contemporary landscape of information technology, the proliferation of interconnected systems and the exponential growth of digital data have brought about unprecedented opportunities as well as formidable challenges. One of the most pressing challenges is to ensure the security of these systems against malicious intrusions that continue to evolve in sophistication and scale. Intrusion detection systems (IDSs) play a pivotal role in fortifying the defenses of networks and computing infrastructures by identifying and thwarting unauthorized access, malicious activities, and anomalies in real-time.

However, the effectiveness of conventional IDS solutions is often hindered by their substantial resource consumption, particularly in terms of energy and computational resources. As concerns about environmental sustainability and energy efficiency continue to mount, there is a growing imperative to develop greener alternatives that mitigate the environmental impact of intrusion detection operations while maintaining high levels of security efficacy.

The sustainability of intrusion detection-based systems in critical sectors like healthcare, smart cities, and defense can indeed be a significant concern due to several key challenges and factors. There are several issues that can lead to unsustainable practices. For instance, IDS can consume significant computational resources, especially if they are inspecting large volumes of network traffic or processing numerous events. This can lead to scalability issues and increased operational costs. In addition, IDSs require regular updates to keep up with emerging threats and vulnerabilities. This involves updating signatures, rules, and policies, which can be time-consuming and resource-intensive.

In addition, IDSs can generate false positives (incorrectly identifying benign activities as malicious) or false negatives (inability to detect actual attacks). Managing these false alerts requires human intervention and can lead to alert fatigue, where security analysts overlook genuine threats amidst a sea of false alarms. In addition, IDSs must adapt to changing threats and network environments. This requires continuous monitoring, tuning, and customization to remain effective against new attack vectors. Depending on the deployment model, IDSs may monitor and analyze sensitive network traffic, raising privacy concerns, especially in environments where user privacy is paramount.

Finally, deploying and managing IDSs can be complex, requiring specialized skills and knowledge. This complexity can deter organizations from implementing IDSs or result in misconfigurations that reduce their effectiveness. Implementing and maintaining IDSs involves significant upfront and ongoing costs, including hardware, software, training, and personnel. Overall, while IDSs are essential for detecting and mitigating cyber threats, their sustainability depends on effectively addressing these challenges through efficient resource utilization, automation, continuous improvement, and alignment with organizational goals and priorities.

**Green intrusion detection systems** (GIDSs) have emerged as a promising paradigm that reconcile the imperatives of security and sustainability. These systems are characterized by their emphasis on minimizing energy consumption, reducing carbon footprint, and optimizing resource utilization without compromising on the core objective of threat detection and prevention. Using innovative techniques from various domains, such as machine learning, data analytics, optimization algorithms, and hardware design, GIDS aims to achieve a harmonious balance between security requirements and environmental responsibility.

This review seeks to answer the central question: *how can green intrusion detection systems effectively integrate sustainability principles without compromising their ability to detect and prevent threats?*

### Our Contributions

In this comprehensive review, we delve into the multifaceted landscape of green intrusion detection systems, exploring their underlying principles, methodologies, applications, and challenges. Through a systematic examination of existing research endeavors and technological advancements, we seek to provide insight into the state-of-the-art approaches toward the development of GIDSs, along with critical assessments of their strengths, limitations, and potential avenues for improvement.

To our knowledge, two previously published review articles exist in the literature [1,2] that specifically review IDSs based on energy optimization. Our current review article differs from theirs in the following ways: (1) our focus is primarily on energy optimization techniques that exist for intrusion detection systems, unlike [1,2] who focused primarily on intrusion detection techniques and secondarily on energy optimization; (2) our survey includes works that were added to the literature after both of the existing reviews.

This review includes the literature covering the following grounds: appropriate study designs that rigorously evaluate IDSs, specific sectors with critical infrastructure (e.g., healthcare, smart cities, and defense) impacted by IDSs, interventions aimed at enhancing sustainability, current practices in IDS deployment and maintenance, and results of interest related to both security efficacy and environmental impact. By addressing these aspects, the review aims to provide a comprehensive understanding of how to develop and implement green intrusion detection systems that align with contemporary demands for both security and sustainability.

Furthermore, we are striving to outline future directions and research opportunities in the field of GIDSs, envisioning innovative strategies and solutions that could propel the evolution of green and sustainable intrusion detection technologies. By fostering interdisciplinary collaboration and knowledge exchange, we aspire to contribute to the ongoing discourse on cybersecurity and environmental stewardship, paving the way towards a more secure, resilient, and environmentally conscious digital ecosystem.

## 2. Generic Energy Optimization Techniques in Cybersecurity

**Figure 1:**
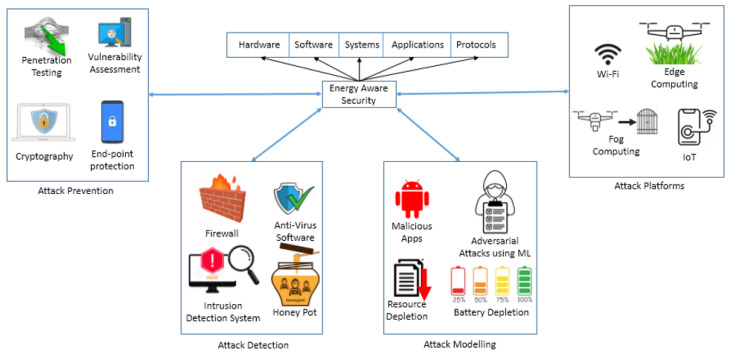
Energy-aware security.

Figure 1 presents a comprehensive landscape of energy-aware security depicted in a pictorial manner. The problem of energy-aware security necessitates the need for a holistic approach to integrate techniques to defend against cyber attacks while minimizing carbon footprint. In particular, novel mechanisms for attack modeling are necessary to understand the ever-increasing capabilities of attacks in diverse forms of networks such as Wi-Fi, cellular, fog and edge computing, and the Internet of Things. In addition, mitigating attacks requires the development of green intrusion detection systems that are capable of increasing detection accuracy while optimizing resources and analyzing the resulting energy–performance–security trade-offs.

In this section, we provide a summary of energy optimization techniques at different layers, such as hardware, hypervisors, operating systems, system software, and applications. In addition, we explore the relationship between energy efficiency in each of the layers and the design of green intrusion detection systems. A summary of the energy optimization techniques at different layers of the computing systems is presented in Table 1.

### 2.1. Energy-Efficient Hardware Selection

A few approaches exist on energy efficient hardware towards designing green intrusion detection systems.

A study [3] examined how much energy intrusion detection software and hardware is used. With a 15-fold increase in throughput, the hardware version only required 0.03% of the energy needed by the software version of the identical algorithm. A different experiment [4] revealed that machine learning algorithms implemented on hardware utilized 46% less energy than their software counterparts, suggesting that energy-efficient hardware implementation is possible.

It was discovered that an anomaly-based intrusion detection technique for embedded systems is both hardware implementation-friendly and energy-efficient [5]. Energy-efficient intrusion detection is crucial to extend the life of wireless sensor networks. The suggested techniques for grouping nodes to carry out intrusion detection are designed to reduce average energy usage and increase network longevity.

Field-programmable gate arrays, or FPGAs, have been investigated for application in intrusion detection. A convolutional neural network based on CPU and FPGA has demonstrated a notable increase in energy efficiency over its software version [6].

Using Xilinx’s Deep Learning Processing Unit IP on a Zynq Ultrascale+ (XCZU3EG) FPGA, Khandelwal et al. [7] offered a lightweight multi-attack quantized machine learning model that was trained and verified using the public CAN Intrusion Detection dataset. With an accuracy of over 99% and a false positive rate of 0.07%, the quantized model detects denial of service and fuzzing assaults with a similar accuracy rate to state-of-the-art methods reported in the literature. With software operations operating on the ECU, intrusion detection system execution uses just 2.0W and delivers a 25% reduction in per-message processing delay over state-of-the-art implementations. The deployment is perfect for real-time IDS in in-vehicle systems because it allows the ECU function to coexist with the IDS with little modification of the tasks.

### 2.2. Energy-Efficient Hypervisors

The foundation of virtualization technology, the hypervisor, optimizes resource consumption by minimizing the total energy footprint and allowing many intrusion detection system instances to run on the same physical hardware. The process of virtualization involves the establishment of virtual instances of memory, storage, and CPUs that are under the supervision of hypervisors. In this section, we look at techniques that help hypervisors run more efficiently and use less energy overall, like hardware-assisted virtualization and power-aware scheduling.

Using power-conscious power consumption profiles, scheduling algorithms [8,9,10,11] seek to maximize virtual machine execution. Hypervisors can lower total power consumption by effectively scheduling workloads with complimentary resource usage patterns. For example, scheduling memory- and CPU-intensive operations concurrently can result in more equitable and effective resource use. Furthermore, these methods emphasize the importance of power-aware scheduling policies for cloud computing systems with high workload variability, with the goal of minimizing energy consumption and ensuring service level agreements (SLAs) while minimizing the system response time [12,13].

Modern CPUs and other hardware have virtualization-supporting characteristics like AMD-V and Intel VT-x. These technologies offer ways to reduce the overhead associated with virtualization, which improves the effectiveness of virtual machine management. Using these hardware-assisted features allows hypervisors to operate at high performance levels with less power consumption [14,15,16]. Thus, it is clear that when considering ARM and MIPS processors, hardware-assisted virtualization is essential for increasing performance predictability and lowering overhead in hypervisors for embedded systems. The advancement of hardware-accelerated hypervisors has also solved issues and greatly enhanced virtualization performance.

### 2.3. Energy-Efficient Operating Systems

Managing the computing system’s resources, such as memory, storage, and computation, is a crucial part of developing operating systems that use less energy. Due to fixed resource budgets, this issue is made worse in resource-constrained systems such as the Internet of Things. Scaling resources up or down in response to demand is the aim of resource management. By scaling resources down during low-load times and up during high-load times, techniques like dynamic power management (DPM) and dynamic voltage and frequency scaling (DVFS) allow systems to consume less energy [17].

By putting components in low-power states while not in use, sleep modes and duty cycling techniques can help intrusion detection systems consume less energy. This method works especially well in settings where network traffic is irregular. Traditionally, energy management in Internet of Things applications has been a hardware support feature. However, with the introduction of low-power modes in current hardware designs, it has become necessary to integrate energy management at the operating system level [17].

In comparison to traditional Linux task scheduling, the energy-aware scheduler (EAS) improves energy efficiency by over 30% by introducing micro-operations performed per joule (OPJ) as a metric for run-time task energy efficiency [18,19]. In order to achieve optimum energy efficiency, a new system software architecture is presented for heterogeneous ISA platforms [37], highlighting the critical role that system software must play in energy reduction.

Reducing operating frequencies and balancing workloads on heterogeneous multicore architectures are two strategies that are the focus of research on energy-efficient mobile computing [20,21,22,23]. However, integrating energy-efficient methods into large-scale computing systems can be difficult. However, new methods such as SYnergy promise to achieve precise energy savings by integrating language, a compiler, runtime, and a job scheduler [24].

### 2.4. Energy-Efficient System Software

Existing research has examined fog and edge computing-based Internet of Things frameworks for energy-efficient system software. Although fog and edge computing are essential for managing the information flow of complex and massive networks, such as the Internet of Things (IoT), their usage can have an effect on electricity prices and carbon emissions [25]. One way to lessen the requirement for data transfer is to deploy IDS functions at the network edge, which is closer to the data source. Localized processing and decision-making are made possible by edge computing, which promotes faster and more energy-efficient processes.

In order to extend network lifespan, research focuses on developing energy-efficient solutions for the edge–fog environment, such as thorough frameworks for energy-efficiency analysis and intelligent energy-management techniques [26,27,28]. In fog computing, task offloading is essential for maximizing resource usage and enhancing system performance. Long-term device functioning and reducing environmental impact require effective energy management [29]. In order to optimize fog node selection and minimize energy usage while respecting service-level agreement (SLA) parameters, studies have presented energy-aware task offloading strategies and dynamic programming approaches [27,29].

In fog computing systems, there are trade-offs between processing and communication resources. Algorithms for figuring out the best resource provisioning to reduce overall energy consumption without compromising service latency performance have been developed based on analytical findings [30].

### 2.5. Energy-Efficient Applications

Energy can be saved at the application level by reducing the volume of data processed by the IDS through the use of adaptive data sampling and filtering techniques. Low energy usage and high detection accuracy are maintained by the system through clever selection and processing of only the most pertinent data.

Roy et al. [31] created a general energy complexity model (ECM) based on double-data-rate synchronous dynamic random access memory (DDR SDRAM) as its reference architecture. DDR’s primary memory is separated into banks, each of which has a set number of pieces. Each bank distributes data in segments. In addition, each bank has a distinct component called the sensory amplifier. For each data access, the necessary data chunk must be brought into the relevant bank’s sense amplifier. Each sense amplifier is limited to holding one chunk at a time, so the current chunk needs to be returned to its bank before a new one is brought in for subsequent access. This model has been applied in different cybersecurity protocols (e.g., [32,38,39,40,41,42]) to optimize their energy consumption.

For smart home contexts, Nimmy et al. [33] created a lightweight authentication protocol based on geometric secret sharing and nonuniformity of the photoresponse (PRNU). The PRNU of a smartphone camera functions as a biometric for distinct identification, eliminating the need to memorize passwords. In comparison to current methods, their suggested system was demonstrated to be both energy-efficient and lightweight. Often, energy consumed by a protocol $E_{protocol}$ is computed using the following equation:

(1) $$E_{protocol}=E_{comp}+E_{comm},$$

where $E_{comp}$ and $E_{comm}$ refer to energy consumed due to computation and communication, respectively.

Protocols perform encryption/decryption along with compression to secure packets, which incur computation energy, and these packets are sent to receiver, thus impacting communication energy. Thus, the energy consumed by computation ($E_{comp}$) and communication ($E_{comm}$) can be further broken down in the following way:

(2) $$E_{comp}=E_{encrypt}+E_{decrypt}+E_{compress},$$

where $E_{encrypt}$, $E_{decrypt}$, and $E_{compress}$ refer to the energy consumed due to operations such as encryption, decryption, and compression, respectively.

(3) $$E_{comm}=E_{transmit}+E_{receive}+E_{idle}+E_{sleep},$$

where $E_{transmit}$, $E_{receive}$, $E_{idle}$, and $E_{sleep}$ refer to energy consumed due to different communication modes such as transmit, receive, idle, and sleep, respectively.

Additionally, Nimmy et al. [34] took advantage of the behavioral traits of Internet of Things (IoT) devices to construct an anomaly detection system utilizing generated data that took into account the effect of malevolent actions like DoS and brute-force attacks on the amount of power that IoT devices consumed. An analysis revealed that behavioral traits such as electricity use have the potential to identify well-known attacks on smart homes.

A lightweight blockchain-based framework for the Narrowband Internet of Things was developed by Mohan et al. [35]. It has features like partitioning dynamic base station memory to improve memory utilization efficiency and scalability and linear hash chain-based storage to avoid costly Merkle tree verification. An analysis of the suggested framework revealed how lightweight it is in relation to current methods. Moreover, to address the shortcomings of the Cu-MAC protocol and enhance channel quality, Mohan et al. [36] created EP-CuMAC for the Narrowband Internet of Things. Furthermore, technologies based on deep learning and machine intelligence are being developed to reduce the effect of retransmissions. The evaluation revealed that, compared to other methods, EP-CuMAC was able to manage the trade-offs between energy, performance, and security.

### 2.6. Lightweight Operating Systems and Virtualization

The open-source operating system Contiki was created mainly with Internet of Things (IoT) devices in mind. It is an operating system that runs on microcontrollers with little resources, including 8- or 16-bit CPUs, and is lightweight, low-power, and memory-efficient. Swedish researcher Adam Dunkels [43] originally released Contiki in 2003 as a component of his doctoral thesis. Since then, its capacity to operate on a variety of hardware platforms and support a number of communication protocols, including IPv6, RPL, and CoAP, has helped it become more well-known in the IoT field.

The framework was built with the Contiki operating system by Arshad et al. [44], who also carried out a thorough study to find any potential performance trade-offs. In order to accomplish effective and economical intrusion detection for IoT systems, the collaborative intrusion detection for IoT (COLIDE) architecture makes use of the basic idea of collaboration between individual sensor nodes and the edge router. An edge router component and a device level make up the framework. The assessment findings show that, while providing effective collaborative intrusion detection for IIoT systems, the suggested framework can decrease energy and communication overheads.

### 2.7. Low-Power Networking Protocols

Nobakht et al. [45] suggested a host-based IDS for smart homes that makes use of software-defined technology (SDN). They suggested the Internet of Things (IoT-IDM) as a framework for intrusion detection and mitigation that would protect smart devices installed in residential settings at the network level. The IoT-IDM monitors the activity of the intended smart device network within the home and looks for potentially harmful or suspicious behavior. This framework is implemented using OpenFlow, an enabling communication protocol for software-defined networking technologies. Ultimately, an IoT-IDM prototype is created, and an actual IoT device, a smart lightbulb, is used to illustrate the applicability and effectiveness of the suggested framework. Various detection modules, such as signature, anomaly, or specification-based approaches, can be used to carry out the detection. The authors assert that putting the intrusion detection module inside an Internet of Things (IoT) device lowers communication cost; however, this also increases the IoT device’s processor overhead, which is important for such low-powered devices.

Using two routing methods, loop-free (LF) KPS and KPS, Ghosh et al. [46] developed an energy-efficient approach for detecting intrusions through unmanned borders and other sensitive sites with prolonged network lifetimes. They demonstrated how data transfer via KPS and LF-KPS protocols will guarantee an extended lifetime for the deployed network by contrasting these two methods with LEACH and TEEN.

An energy-efficient security-aware architecture for wireless control systems intended for application in factory automation was proposed by Muradore et al. [47]. They suggested using packet-based selective encryption to cut down on energy usage and identify the beginning and end of an assault. Since the packet transmission rate affects energy usage as well, particularly during attacks, they advised tailoring the approach to the performance of instantaneous control.

The rest of the paper is organized in the following manner. In Section 3, certain important terms related to intrusion detection and energy efficiency are defined. Section 4 contains the relevant reviews that looked at intrusion detection methods for energy consumption and optimization. Section 5 presents the proposed taxonomy and a review of the literature on IDSs for energy optimization. Section 6 offers one of the work’s most significant contributions: a thorough analysis of open issues and possible directions for further research on IDSs in energy optimization. Finally, in Section 8, we provide some concluding remarks.

## 3. Intrusion Detection Deployment and Techniques

This section reviews modern intrusion systems generically. IDSs are categorized on the basis of functionality. Table 2 summarizes different kinds of IDS techniques and their corresponding deployments in numerous environments.

### 3.1. Intrusion Detection Deployment

**Figure 2:**
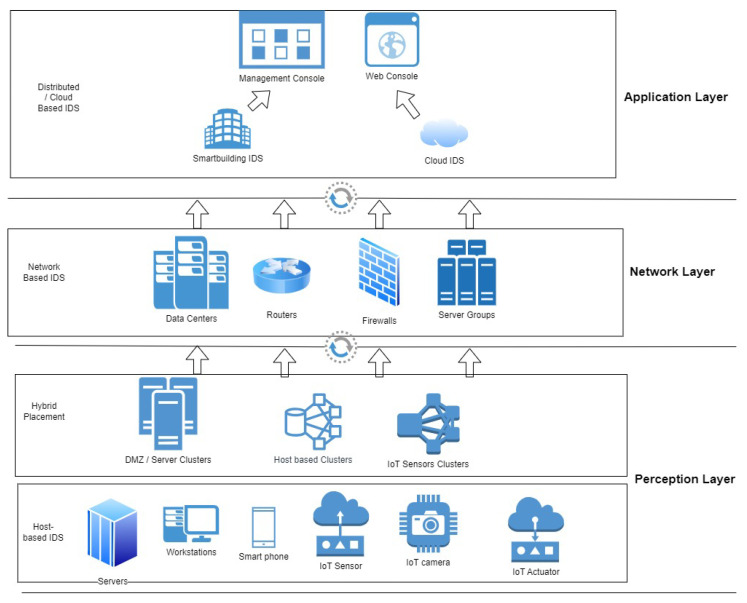
IDS deployment.

Intrusion detection systems (IDSs) play a crucial role in securing various environments by monitoring and analyzing activities to detect malicious behaviors. Figure 2 illustrates the deployment of IDSs in three layers: the application layer, the network layer, and the perception layer. An IoT IDS focuses on the specific needs of IoT ecosystems, employing lightweight protocols and algorithms to efficiently monitor IoT devices’ traffic and behavior, detecting anomalies or unauthorized access attempts. A host-based IDS (HIDS) operates on individual devices, monitoring system calls, application logs, and file system modifications to detect suspicious activities, providing detailed information on potential threats targeting specific hosts. HIDSs can be installed on a single host or configured across a cluster of hosts, with the IDS installed on a centralized server or the cluster’s primary node, a configuration known as hybrid placement. A network-based IDS (NIDS) monitors network traffic for suspicious patterns across the entire network, using methods like signature-based detection and anomaly detection to identify potential threats, ensuring a broad scope of network security. A cloud IDS is customized for cloud environments, using native cloud tools and services to monitor virtualized resources, data flows, and user activities within the cloud infrastructure, ensuring scalable and efficient threat detection in dynamic cloud environments. Each type of IDS addresses unique security challenges, contributing to a comprehensive defense strategy. The following subsections will discuss the different categories of IDS deployment in detail.

#### 3.1.1. IoT Intrusion Detection

Sarika et al. [48] and Zarpelao et al. [49] offer an overview of the Internet of Things, including its security threats and different intrusion detection methods. Three layers, the application layer, network layer, and perception layer, summarize the architecture of IoT systems by Sarika et al. Placement strategies for IDSs in the Internet of Things were divided into three categories by Zarpelao et al. [49]: distributed, centralized, and hybrid. An IDS is positioned in each physical component of the LLN (low-power and lossy network) as a distributed strategy. These are self-contained nodes with optimized IDSs installed in each [50,51]. The nodes in the distributed placement may also be in charge of keeping an eye on their neighbors. A router, server, or dedicated host are examples of centralized components where the IDS is positioned in a centralized configuration. Every piece of information collected and sent to the Internet by the dispersed nodes passes through the central device. Consequently, all of the traffic exchanged may be analyzed by the IDS installed in the centralised device [52,53]. In order to capitalize on their advantages and minimize their disadvantages, hybrid IDS placement integrates the ideas of dispersed and centralized deployment. One method is to divide the network into clusters and only allow each cluster’s primary node to host on an IDS [54,55,56].

#### 3.1.2. Network Intrusion Detection

A network-based intrusion detection system (NIDS) is a tool used to watch and examine network traffic in order to find malicious activity or policy infractions. An NIDS examines all of the data moving via a network segment. Unauthorized access attempts, virus propagation, denial-of-service attacks, and other threats can be detected by an NIDS by examining packets for known attack patterns, unusual behaviors, and policy violations. It frequently uses anomaly-based detection (Section 3.2) for novel or unidentified threats and signature-based detection Section 3.3.5) for known threats. To provide complete visibility and protection throughout the network architecture, an NIDS can be placed at key network locations, such as the on perimeter of the network or within particular network segments (Figure 2). Due to its real-time threat detection and response capabilities, it is a crucial part of any effective cybersecurity plan.

Various machine learning, data mining, artificial intelligence, and statistical techniques are available for anomaly-based network intrusion detection systems (NIDSs). The specifics of them will be covered in Section 3.2. One of the newest and most popular methods for identifying anomalies is deep learning. For unsupervised feature learning, Shone et al. [57] presented a deep learning classification model on an NIDS built using a stacked non-symmetric deep autoencoder. Their classifier was constructed in TensorFlow, a graphics processing unit (GPU) capable framework, and tested on the NSL-KDD and KDD-Cup’99 benchmark datasets. An effective and adaptable NIDS can be developed using the deep learning-based method suggested by Niyaz et al. [58]. They also used NSL-KDD datasets for self-taught learning (STL), a deep learning-based approach. Vianyakumar et al. [59] used millions of known good and bad network connections to train supervised learning methods like multilayer perceptron (MLP), CNNs, CNN-recurrent neural networks (CNN-RNNs), CNN-long short-term memory (CNN-LSTM), and CNN-gated recurrent units (GRUs) to model network traffic as time series, specifically transmission control protocol/internet protocol (TCP/IP) packets within a predetermined time range.

#### 3.1.3. Host-Based Intrusion Detection

The purpose of a host-based intrusion detection systems (HIDSs) is to identify indications of malicious activity or policy violations by tracking and analyzing activities on specific devices or hosts. An HIDS offers an in-depth perspective of the security status of every host it safeguards through the analysis of system logs, file integrity, and process activities. With its concentration on the internal operations of a single device, an HIDS is more successful than a network-based IDS at spotting insider threats, illegal modifications, and local attacks. A network-based IDS, on the other hand, monitors traffic throughout the network. It can use signature-based detection to identify known attack patterns or compare the current behavior of the system to a baseline of typical activity to identify abnormalities [60]. When an HIDS detects questionable activity, including unwanted access attempts, modifications to important files, and odd system operations, it can send notifications. It is an essential line of defense for guaranteeing the integrity and security of individual systems inside an organization’s overall security framework because of its comprehensive, host-specific monitoring capabilities.

Anomaly detection techniques are also widely utilized in HIDSs; an agent-based artificial immune system (ABAIS) was introduced by Ou Chung-Ming [61] into the HIDS. The risk hypothesis of the human immune system serves as the inspiration for the proposed agent-based IDS (ABIDS). An ABIDS has multiple embedded agents that work together to update the activation threshold for security responses and compute the mature context antigen value (MCAV). An anomaly detection method based on the semantic interactions of system calls was presented by Syed et al. [62]. The main idea is to model system calls as kernel module states, examine state interactions, and compare the odds of various state occurrences in anomalous and normal traces to find anomalies. By using this method, one can have a visual comprehension of the behavior of the system and make better decisions.

#### 3.1.4. Cloud Intrusion Detection

Due to the increased use of cloud environments over the past ten years, there has been a demand for cloud intrusion detection. Strong security measures like cloud intrusion detection systems (CIDS) are essential as more and more businesses move their infrastructure and services to the cloud. Their purpose is to safeguard cloud environments from hostile activity, illegal access, and data breaches. In order to detect any attacks in real-time, these systems track and examine user activity, system behaviors, and cloud network traffic. Cloud intrusion detection systems offer full visibility and protection in public, private, and hybrid cloud environments when coupled with the native security tools of cloud service providers and/or third-party security solutions (Figure 2). A CIDS minimizes the risk of data loss and preserves the availability and integrity of cloud resources by providing fast notifications and comprehensive forensic information to enterprises.

Palo Alto Networks powers the Google Cloud intrusion detection system (Cloud IDS), which provides network-based threat detection with signature-based detection capabilities [63]. It is intended to provide comprehensive context for security events in Google Cloud environments and to identify malware and intrusions.

GuardDuty from Amazon Web Services (AWS) is a managed threat detection service that looks out for unusual or harmful activity. It identifies and ranks possible risks in AWS accounts, workloads, and data stored on Amazon S3 using machine learning, anomaly detection, and integrated threat intelligence [64].

Advanced threat protection for hybrid cloud workloads is another feature provided by the Microsoft Azure Security Center. It offers comprehensive security monitoring and policy management across Azure subscriptions, using analytics and machine learning to identify and address threats.

### 3.2. Anomaly-Based Intrusion Detection Techniques

Machine learning algorithms are used by anomaly-based intrusion detection systems (IDS) to detect changes from a system, network, or user’s typical behavior. These are a few typical uses for anomaly IDS machine learning methods.

#### Deep Learning

Nie et al. [65] provided a deep reinforcement learning-based intrusion detection method that first used statistical aspects of historical network traffic to forecast traffic patterns. Next, they applied intrusion detection using traffic predictors. The tests confirm our algorithm’s ability to identify distributed denial-of-service (DDoS) assaults. The suggested model combined machine learning and intrusion detection systems to increase the precision of green IoT intrusion detection.

Using deep learning for anomaly-based intrusion detection systems (IDSs) to secure IoT environments, a systematic literature review by Alsoufi et al. was developed [50]. It talks about supervised versus unsupervised learning, how effective deep learning approaches are, and offers insights into the examination of previous research in this field.

In Alrawashdeh et al. [66], deep belief networks and the restricted Boltzmann machine (RBM) are the main topics of discussion while using a deep learning strategy for anomaly detection. The DARPA KDD-Cup’99 dataset will be used to evaluate the architecture, performance, and potential options for expanding the approach’s application to bigger datasets.

### 3.3. Outlier Detection

In their article [67], Jabez and Muthukumar described how they used outlier detection to find anomalies in an IDS. They explained the methodology, which includes the use of the neighborhood outlier factor (NOF), and showcased the experimental findings that demonstrate how successful the suggested strategy is.

#### 3.3.1. KNN

A suggested anomaly-based intrusion detection system (IDS) by Chordia and Gupta [68] uses data mining approaches to lower false alarm rates and improve detection efficiency. With a focus on U2R, R2R, DoS, and probe attacks, the suggested system employs techniques like K-NN, K-means, and decision table majority rule-based methodology to monitor network traffic. The authors evaluated the effectiveness of the approach using the KDD 99 dataset, which emphasizes the lack of security event data from an IoT system to support a more thorough and balanced assessment of IDS systems for IoT.

#### 3.3.2. Naive-Bayes

In comparison to an analogous software (SW) version, Viegas et al. [5] showed that a hardware (HW) implementation of network security algorithms can drastically reduce their energy usage. They built an anomaly-based network intrusion detection system (NIDS) using three machine learning (ML) classifiers implemented in SW and HW-decision tree (DT), Naive-Bayes (NB), and k-nearest neighbors (kNN). They suggested a new feature extraction approach with minimal processing needs and hardware implementation compatibility. The new feature extractor used a lot less memory, electricity, and processing power. Its HW implementation used only 12% and its SW implementation only 22% of the energy of a commercial device. Energy savings of up to 93% were made possible by dual-objective feature selection.

#### 3.3.3. Statistical Model

According to Riecker et al. [69], a system that is energy-efficient and lightweight utilizes mobile agents to identify intrusions by measuring the energy usage of sensor nodes. An energy consumption prediction model based on linear regression was utilized. According to simulation studies, flooding and other denial-of-service attacks can be identified with a high degree of precision and a very low rate of false positives.

#### 3.3.4. New Anonymous Detection Model

Since sensor networks are constructed for different intruders in different scenarios, Chen et al. [70] examined the detection probability of an arbitrary path across the barrier of sensors theoretically and took into account the maximum speed of conceivable intruders. They provided an energy-efficient scheduling problem for sensors by formulating a minimum weight ϵ-barrier problem based on the theoretical study of detection probability. In order to schedule the activation of sensors, they demonstrated that the problem is NP-hard and suggested a bounded approximation approach known as the minimum weight barrier approach (MWBA). In order to assess our design, they run comprehensive simulations to show the efficacy of our suggested algorithm in addition to conducting a theoretical analysis of MWBA performance.

The intelligent intrusion detection system Passban IDS [71] is intended for Internet of Things edge devices. It demonstrates how important it is to secure Internet of Things devices, how to use Passban on low-cost IoT gateways, and how it can identify different kinds of malicious traffic with low false-positive rates.

A low-complexity, energy-conscious approach for intrusion detection in wireless sensor networks was proposed by Misra et al. [72]. The protocol includes distributed and self-learning. When one node is compromised, the dispersed nature prevents all other nodes from being sacrificed. The protocol aims to create an intrusion detection system that is mindful of energy by contrasting the idea of stochastic learning automata with the packet sampling mechanism.

Border intrusion detection was proposed [73] by Yang et al. It provides an energy-efficient way for border patrol to increase detection accuracy while lowering the heavy human engagement. In addition, they created a brand new coverage model to identify one-way paths. According to the simulation results, the new coverage model has the ability to efficiently extend the network life and identify intrusions in border areas.

#### 3.3.5. Signature-Based Intrusion Detection Techniques

Due to its open source nature and widespread use in the intrusion detection and prevention space, Snort is mostly utilized as a signature-based intrusion detection system. Snort warnings are also viewed using the Basic Analysis and Security Engine (BASE) [74,75].

According to Nattawat et al. [76], the Snort-IDS rules for the detection of network probe attacks can be improved. Another signature-based network intrusion prevention (NIPS) and network security (NIDS) engine is called Suricata. Its purpose is to monitor network traffic for potentially dangerous activity and suspicious activity, giving businesses the ability to strengthen their cybersecurity defenses. It can decode a wide range of protocols, examine network packets at different layers, and use both signature-based and anomaly-based detection methods to find anomalies. The processing and detection rates of Snort and Suricata were examined and contrasted by Wonhyung Park and Seongjin Ahn [77] in order to debate which is superior in environments with a single thread or many threads.

An automated machine learning architecture for Internet of Things (IoT)-enabled smart energy grids that can determine whether to develop rules for signature-based systems was proposed by Yadav et al. [78]. The framework’s potential for intelligent threat mitigation in smart energy infrastructures was demonstrated by the results, which were obtained using an IoT dataset that included MITM (man in the middle) assaults.

A dynamic coding approach was presented by Amin et al. [79] to assist in the implementation of an intrusion detection system (IDS) based on distributed signatures in IP-USNs (IP-based ubiquitous sensor networks). The suggested plan is suitable for resource-constrained sensor devices as it allows the construction of lightweight IDSs in terms of messaging, storage, and energy usage.

The focus of Bostani and Sheikhan [80] was on a brand-new real-time hybrid intrusion detection system suggested for the Internet of Things. It highlights the deployment of anomaly-based and specification-based intrusion detection modules in Internet of Things situations, as well as the performance evaluation of the suggested framework.

### 3.4. Hybrid of Signature-Based IDS and Anomaly IDS

In their paper [81], Echateerawat et al. compared various methods for detecting intrusions in sensor networks. We examined the relationship between energy efficiency and assault detection accuracy. They proposed that the greatest features might be combined by creating a hybrid system that combines anomaly and signature IDSs.

The architecture of the hybrid intrusion detection system (eHIDS) for wireless sensor networks was proposed by Abduvaliyev et al. [82]. They used a combination of anomaly and signature-based detection techniques to create a hybrid scheme. In addition, they employed cluster-based wireless sensor networks to reduce the cost of computing and communication. They simulated the network and compared the performance of our scheme with that of other similar methods. The technique outperformed other schemes in terms of the high detection rate and energy efficiency, according to the simulation findings.

Tama et al. [83] and Rizzardi et al. [84] presented an enhanced IDS that uses two-level classifier ensembles and hybrid feature selection. The technique, dataset performance evaluation, and the importance of statistical significance tests in confirming the findings were covered.

An analysis of the suggested anomaly detection technique for supervisory control and data acquisition (SCADA) systems was provided by Bostani and Sheikhan [80]. Preprocessing methods, dimensionality reduction algorithms, dataset balance, and experimental findings demonstrating the effectiveness of the suggested strategy were all covered.

**Table 2:** Summary of IDS deployment and techniques.

		IoT IDS	Host-Based IDS	Network-Based IDS	Cloud-Based IDS
**Signature-based** **IDS Technique**		Yadav, N. et al. [78]	Liu, M. et al. [60], Murtaza, S.S et al. [62]	Kumar, V. et al. [75], Kurundkar, G. et al. [74], Khamphakdee, N. et al. [76], Park, W. et al. [77]	
**Anomaly** **IDS** **Techniques**	**Deep** **Learning**	Oh, D. et al. [50], Nie, L. et al. [65]		Shone, N. et al. [57], Javaid, A. et al. [58], Alrawashdeh, K. et al. [66]	
	**Outlier ** **Detection** **KNN**	Gupta, S. et al. [68]		Jabez, J. et al. [67]	
	**Naive-Bayes**			Viegas, E. et al. [5]	
	**Supervised** **Statistical ** ** Model**	Riecker, M. et al. [69]	Vinayakumar, R. et al. [59]		
	**New** **Anonymous** **Detection**	Chen, J. et al. [70], Eskandari, M. et al. [71], Misra, S. et al. [72], Yang, T. et al. [73]	Ou, C.M. [61]		
**Hybrid of Signature** **& Anomaly IDS**		Yadav, N. et al. [78], Bostani, H. et al. [80], Techateerawat, P. et al. [81], Abduvaliyev, A. et al. [82]		Tama, B.A. et al. [83]	Google Cloud IDS [63], Amazon GuardDuty [64]

## 4. Energy Optimization Techniques in Intrusion Detection Systems

In this section we briefly mention the techniques used in recent IDSs to optimize energy consumption. We emphasize energy optimization techniques over detection mechanisms in this section.

Migliardi et al. [85] suggested making an effort to evaluate the energy impact of security measures. Specifically, they offered a basic model for assessing the energy cost of distributed packet inspection in intrusion detection systems (IDSs) and demonstrated how to apply it to two example IDS tactics to assess energy leakage resulting from the late identification of rogue packets.

Arshad et al. [86] designed a framework called collaborative intrusion detection (COLIDE) for IoT networks. The framework specifically allows for the combined use of data from network-based detection systems and hosts. The end-host/node layer and the edge router layer make up the two tiers of the detection system. In order to correlate the warnings and carry out aggregate detection, the end-host component keeps an eye on events at the node level and communicates aberrant events to the network/edge router level system.

They claim that by coordinating alerts from many devices, the workload at the end host will be decreased in addition to minimizing false positive rates and enhancing detection rates under spread attacks. As a result, issues like flexibility, node resource limitations, and the collaborative character of IoT networks were anticipated to be addressed by the suggested framework.

In their experiments power measurements were performed with Contiki OS’s powertrace utility [87]. Their simulation results indicate that a node needs approximately 5 mW of power to process 1000 packets, which is insignificant for the ultra-low-power Tmote sky [88] in terms of energy overheads.

Wang et al. [89] provided an attack–defense game model to identify malicious nodes using a repeating game technique, with an emphasis on intrusion detection approaches. To obtain the best payoffs in the suggested game model, attackers and defenders adopt various techniques.

Machine-to-machine (M2M) mobile networks must be extremely dependable since the devices with computational capabilities in them use data that have been acquired to compute things that are physical and then provide the results to other devices. In order to guarantee that the system can function as intended, the defense system for sensor network (SN) security in M2M mobile networks must adapt its reactions to various attack vectors. In their work [89], they present the use of game theory as a tool for designing an attack–defense game model, with the goal of determining the optimal attack and defense strategies through repeated game methods.

A repeating game model is suggested as a solution to M2M mobile network intrusion detection issues. To help M2M mobile networks analyze and determine the best tactics for attackers and defenders, a game tree model is proposed. To assess how well their model performs in comparison to two other models (all monitor (AM) and cluster head (CH)) that are currently in use, simulation is carried out.

Their attack–defense game model, which is based on game theory, almost always uses less energy than the AM and CH models. In particular, when compared to the AM model, the game theory-based attack defensive game model can save up to 50% on energy usage.

Sedjelmaci et al. [90] suggested using game theory to activate anomaly detection methods solely in anticipation of a new attack’s signature; this achieves a balance between energy consumption, false positive rates, and detection rates. According to simulation results, this lightweight anomaly detection method works better than existing anomaly detection techniques because it uses less energy in scaling mode (i.e., when there are a lot of IoT devices and attackers) to detect attacks with high detection and low false-positive rates. The energy efficiency is achieved by a need-based invoking of more energy consuming anomaly detection based on a game theoretic approach (over the lower energy consuming signature detection) in intrusion detection systems.

Raza et al. designed, built, and assessed SVELTE, a novel intrusion detection system for the Internet of Things, in their article [91]. They mainly focused on routing threats such spoofing or altering information, sinkholes, and selective forwarding in their installation and assessment. Their method can be expanded to identify other attacks. They integrated SVELTE into the Contiki OS and conducted a comprehensive assessment. According to their assessment, SVELTE is able to identify any malicious node that initiates its implemented sinkhole and/or selective forwarding assaults in simulated scenarios. Nevertheless, there are some false alarms generated when malicious nodes are detected, meaning that the genuine positive rate is not 100%. Furthermore, SVELTE may be deployed on constrained nodes with low energy and memory capacities due to its negligible overhead.

Earlier techniques for energy optimization in IDSs have involved cutting down on communication overheads [92,93,94,95], cutting down on computational overheads [96,97,98,99,100], or dividing up in-network tasks [101,102].

## 5. Taxonomy of Green IDS Techniques

We used a comprehensive set of criteria and metrics to carry out a complete and methodical review of the current literature, which is described in Section 3 and Section 4. These metrics are important for efficient energy optimization in intrusion detection systems. The criteria’s individual components are listed below, each with a brief explanation. Table 3 presents a comparative examination of current techniques for these criteria.

- **Architecture.** An intrusion detection system’s architecture details how the detection system performs detection tasks. User privacy is impacted by the system’s design in addition to performance and detection accuracy. Because the standalone detection system primarily functions on a local machine or device, it is vulnerable to longer detection times due to insufficient data availability and the stealthy character of the attacker. In contrast, a collaborative architecture makes use of data from several sources, such network devices or Internet of Things devices, whether they are part of the same company or not. It can increase the accuracy of detection, but it also raises concerns about the privacy of data shared between entities. In addition, an edge router that controls communication between the local network and the Internet and a number of IoT devices arranged into a local network, e.g., 6LoWPAN, make up a typical IoT system in terms of energy optimization and detection accuracy.
- **Detection technique.** As mentioned in Section 3 and Section 4, an IDS can make use of a range of detection methods, including anomaly, signature, and game-based techniques. The selection of a detection engine affects an intrusion detection system’s (IDS) capacity to identify attacks as well as the energy consumption of the system. For example, while signature-based intrusion detection systems (IDS) have been found to be energy-efficient, they are unable to identify zero-day assaults. In order to achieve effective intrusion detection, an increasing amount of artificial intelligence and deep learning is being used according to an analysis of the literature that is presented in Section 3 and Section 4 and summarized in Table 3.
- **Energy optimization technique.** The technique(s) used for energy optimization in the illustrated IDS are highlighted in this review. As mentioned before, the primary objective of our paper is to review the existing energy optimization techniques for IDSs and present our ideas on the potential application of other existing energy optimization techniques onto modern IDSs for further energy optimization.

## 6. Future Research Directions

Table 3 reveals the following: (1) not many works exist in the literature that specifically treat energy as a first-class parameter to optimize IDSs; (2) most of energy-optimization techniques have been achieved as a byproduct of making detection more efficient in IDSs.

Specifically, to our knowledge, no work has been carried out to *engineer existing IDS algorithms* to optimize energy consumption in them (e.g., applying the ECM of [31]). In Table 4, we provide a mapping of *potential* energy optimization techniques from Section 2 on the IDS systems of Section 3.

As illustrated in Table 4, potential energy-optimization techniques have been identified that can be applied to the modern IDSs. This table was created to serve as a thinking point for future researchers. In the remainder of the section, we provide our thoughts on how existing energy-optimization techniques potentially apply on the modern IDSs.

### 6.1. Energy Complexity Model (ECM) [31]

As mentioned in Section 2, the ECM proposed by [31] optimizes energy consumption in algorithms by engineering them to ensure parallel memory bank accesses. In theory, with a *P* bank DDR3 architecture with *B* bytes per chunk, the energy consumed by an algorithm A with the execution time τ is given by a [31]-derived formula:

(4) E(A)=τ+(P×B)/I.

For each *P* block access made overall, the so-called *parallelization index*, denoted by *I*, is essentially the number of parallel block accesses performed by A across various memory banks. According to ECM, an algorithm’s ability to reduce energy consumption is inversely correlated with how well it can be designed to parallelize memory access. Furthermore, algorithms that process data in *blocks* have more potential to be engineered using ECM for energy optimization. These algorithms are called *block structured* in [31].

Shone et al. [57] and Niyaz et al. [58] used deep learning algorithms for intrusion detection, which are block structured by design. One of the central algorithms, the auto-encoder, accepts inputs in a natural block-structured form on which the ECM [31] can be potentially applied.

### 6.2. Reducing Operating Frequencies and Balancing Workloads

Research on energy-efficient mobile computing focuses on two strategies: lowering operating frequencies and balancing workloads on heterogeneous multicore architectures [15,16,18,19]. The techniques based on supervised learning methods (e.g., [59]), that in order to train supervised learning techniques such as multilayer perceptron (MLP), CNN, CNN-recurrent neural network (CNN-RNN), CNN-long short-term memory (CNN-LSTM), and CNN-gated recurrent unit (GRU) to model network traffic as time series, specifically transmission control protocol/internet protocol (TCP/IP) packets within a predetermined time range, use millions of known good and bad network connections.

### 6.3. Software-Defined Network (SDN)

By relocating fog nodes and creating fewer fog servers, the fog layer seeks to minimize the number of active fog servers [27]. Given a fog node placement matrix A, let it be described by

$$A_{ij}=\left\{\begin{array}{cc} 1 & if\;fog\;node\;j\;is\;placed\;on\;fog\;server\;i,\\ 0 & otherwise. \end{array}\right.$$

The demand of fog node *j*’ for the accessible resource type *r* is represented by Nrj, while Fri represents the resource type *r* currently available on fog server *i*. Let FSi be a binary variable that takes on the value 1 while the fog server *i* is active and 0 otherwise.

(5) $$Minimize\;\sum_{i}FSi,$$

(6) $$Subject\;to\;\sum_{j=1}Nrj\times Aij\leq Fri\;\forall i,r$$

(7) $$\sum_{i}A_{ij}=1\;\forall j,$$

(8) $$FSi\geq A_{ij}=1\;\forall i,j,$$

where 1≤i≤|F|, 1≤j≤|N|, and r∈ℜ.

The number of fog servers that are turned on is minimized by the objective function (5). The second limitation (6) states that the total resource demands installed by the fog nodes on a particular fog server cannot exceed the fog server’s capability. Every fog node must be installed on precisely one fog server according to Constraint (7). Equation (8) uses the variables $FS_{i}$ and $A_{ij}$ to track whether a fog server is in operation and turns it on or off.

Ou Chung-Ming [61] designed a host IDS (HIDS) based on an agent-based artificial immune system (ABAIS), having multiple embedded agents working together to update the activation threshold for security responses and compute the mature context antigen value. The ABAIS HIDS algorithm works based on an *antigen–signal* pair, solving a similar objective function described below.

If the distance between an antigen–signal pair (A,S) and its threshold vector $(T_{h}\left(I\right)$ of *I* is equal to ε, the computer host *I* is said to have experienced an ε-intrusion, $d(TP\left(S\right),T_{h}\left(I\right))=\varepsilon$. The following is the definition of *distance* (*d*):

(9) $$d\left(x,y\right)=\sum_{i=1}^{3}max\left(x_{i}-y_{i},0\right),$$

where $x=(x_{1},x_{2},x_{3})$, $y=(y_{1},y_{2},y_{3})$.

It is critical to define a valid value ε in order for ε-intrusion to qualify as a dangerous attack type. Reducing false-positives and false-negatives of IDSs is also indicated by the correct value for ε.

The similarities in objective functions in Equations (5) and (9) lead us to believe that the SDN-based energy optimization techniques in [27] can be applied to the ABAIS-based HIDS in [61]. This has been listed in Table 4. Syed et al.’s [62] kernel states modeling (KSM) approach exhibits a similar potential application of [27] to reduce energy consumption.

### 6.4. Power-Aware Scheduling Algorithms

Power-aware scheduling methods use power consumption profiles to optimize virtual machine execution. Hypervisors can reduce overall power consumption by efficiently allocating workloads to complementary patterns of resource utilization. Concurrent scheduling of memory- and CPU-intensive tasks, for example, can lead to more equitable and efficient resource consumption. The aforementioned techniques underscore the significance of power-aware scheduling algorithms in cloud computing systems that exhibit significant workload unpredictability [8,9,10,11,12,13]. The objective is to minimize energy usage and maintain service-level agreements (SLAs) while simultaneously reducing system response times. Therefore, it will be interesting to see how these techniques apply to reduce energy consumption in cloud-based IDSs (e.g., Google [63] and Amazon [64]).

### 6.5. A Lightweight Blockchain-Based Framework for Networks

Since the sensor networks are built for various intruders in various conditions, Chen et al. [70] considered the maximum speed of potential intruders and theoretically analyzed the detection probability of an arbitrary path across the sensor barrier.

Mohan et al. [35] created a thin, blockchain-based framework for the Narrowband Internet of Things. Its characteristics include linear hash chain-based storage to prevent expensive Merkle tree verification and dynamic base station memory partitioning to improve memory use efficiency and scalability. The lightweight nature of the suggested framework in comparison to existing techniques was discovered through a study.

Mohan et al.’s [35] work was specifically targeted towards energy optimization in sensor networks. Therefore, Chen et al.’s [70] IDS presents the potential for the application of Mohan et al.’s techniques for further energy optimization.

## 7. Limitations of This Review

While this review provides a comprehensive analysis of green intrusion detection systems (GIDS) and their application in various critical sectors, several limitations should be acknowledged.

Firstly, we did not rank the quality of the literature reviewed nor discuss the funding sources used in the studies. Although we provided four taxonomy tables summarizing the relevant research in different categories, we plan to include a ranking and discussion of the ranking criteria in future studies to enhance the robustness and reliability of our findings.

Secondly, due to paper length restrictions, we were unable to include discussions, tables, or charts detailing the studies included in the review regarding sample sizes, populations studied, time frames of studies, missing data, limitations, outcome measures, and final results. Instead, our tables focus on author information, interventions, IDS techniques used, algorithms, and architectures of IDS systems. The absence of these detailed summaries may limit the ability to fully assess the comparative strengths and weaknesses of the studies reviewed.

Furthermore, while we have thoroughly discussed the sustainability aspects and effectiveness of GIDSs, the exclusion of detailed rankings and funding source discussions could limit the contextual understanding of the impact and potential biases of the reviewed studies.

In summary, despite these limitations, this review offers significant insights into the development and implementation of sustainable intrusion detection systems. We are confident that future research will address these gaps by incorporating detailed rankings, funding source analyses, and comprehensive study summaries to provide a more thorough evaluation of the GIDS literature.

## 8. Conclusions

In this paper, we conducted a comprehensive review of energy-optimization techniques applied to modern intrusion detection systems (IDS). The proliferation of Internet of Things (IoT) systems has significantly increased the volume and variety of security risks, highlighting the necessity of effective IDSs. Given the high energy consumption associated with most IDSs, optimizing energy usage has become a critical consideration. Our review examined state-of-the-art energy optimization strategies for contemporary IDSs, identifying potential applications of general energy-optimization approaches. Consequently, this study outlines future research directions for developing energy-efficient IDSs, particularly in the context of deep learning-based systems. Therefore, we believe that the work has laid out future research routes to build energy-efficient intrusion detection systems. Of special interest will be performing a similar energy-optimization analysis on generic deep learning-based systems (e.g., [103]).

## Figures and Tables

**Table 1:** Summary of existing approaches for energy optimization.

Layer	Techniques	Papers
Hardware	Hardware Acceleration	[3,4,5,6,7]
Hypervisors	Virtualization and Containerization	[8,9,10,11,12,13,14,15,16]
Operating Systems	Resource Management	[17,18,19,20,21,22,23,24]
System Software	Task Offloading	[25,26,27,28,29,30]
Applications	Adaptive Sampling and Filtering	[31,32,33,34,35,36]

**Table 3:** Energy optimization techniques for different IDSs (green column lists energy-optimization techniques).

IDS	Architecture	Energy Optimization Technique	Detection Technique	Other Features
Migliardi et al. [85]	Generic NIDS	Energy Leakage Reduction	Distributed Intrusion Detection Scheme	Novel energy modeling of DIDS
Arshad et. al [44]	6LoWPAN	Energy Optimized via Coordinating Alerts from many Devices	Collaborative Intrusion Detection	Can be applied to other routing protocols
Wang et al. [89]	Machine-to- Machine (M2M) mobile Networks	Game Tree Model for finding Optimal Strategies	Attack-defense Game model	Can reduce energy consumption by up to 50% in comparison to similar IDS
Sedjelmaci et al. [90]	Wireless Sensor Networks	Need-based invoking of Anomaly Detection	Game Theoretic Approach	Low false positive rates
Raza et al. [91]	6LoWPAN	Dependent over implementation on low-power networks	IDS integrated with Mini Firewall	True positive rate is not 100% (some false alarms present)
Viegas et al. [4]	Generic NIDS	Hardware (HW) Implementation of Network Security Algorithms	Anomaly based NIDS with Machine Learning Classifiers implemented in SW and HW-Decision Tree, Naive-Bayes and k-Nearest Neighbors.	SW implementation consumes only 22% of the energy used by a commercial product and its HW implementation only 12%.
Riecker et al. [69]	Generic NIDS	Linear Regression model is Applied to Predict the Energy Consumption	Mobile Agents to Detect Intrusions based on Energy Consumption of Sensor Nodes	Denial-of-Service attacks detected with high accuracy, and low false positives

**Table 4:** Potential energy optimization techniques for existing IDS (green column lists energy-optimization techniques).

IDS	Architecture	Potential Energy Optimization Technique	Detection Technique
Shone et al. [57]	GPU-enabled Tensorflow	Energy Complexity Model (ECM) [31]	Deep Learning Classification Model
Niyaz et al. [58]	GPU-enabled Tensorflow	Energy Complexity Model (ECM) [31]	Self-taught Learning (Deep Learning based Approach)
R Vinayakumar et al. [59]	Generic NIDS	Reducing Operating Frequencies and Balancing Workloads [15,16,17,18,19]	Supervised Learning Methods (e.g. CNN)
Ou Chung-Ming [61]	Host Based	Software Defined Network (SDN) [27]	Agent-based Artificial Immune System
Syed et al. [62]	Host Based	Software Defined Network (SDN) [27]	Semantic Interactions of System Calls
Google Cloud Intrusion Detection System [63]	Cloud IDS	Power-Aware Scheduling Algorithms [8,9,10,11,12,13]	Signature-based Detection
Amazon Web Services (AWS) GuardDuty [64]	Cloud IDS	Power-Aware Scheduling Algorithms [8,9,10,11,12,13]	Machine Learning, Anomaly Detection
Chen et al. [70]	Network Sensors	Lightweight Blockchain Based Framework for Networks [35]	Minimum Weight Barrier Algorithm (MWBA)
Chordia and Gupta [68]	Generic NIDS	Energy Efficient Wireless Control Systems [89]	Anomaly-Based IDS
Jabez and Muthukumar [67]	Generic NIDS	EP-CuMAC for Narrowband Internet of Things [36]	Neighborhood Outlier Factor
